# Cobalt‐Based Catalysts for Electrochemical Water Splitting: Harnessing the Power of Ionic Liquids and Deep Eutectic Solvents

**DOI:** 10.1002/open.202500423

**Published:** 2026-03-02

**Authors:** Jiahao Wang, Chenyun Zhang

**Affiliations:** ^1^ Wuxi Vocational Institute of Arts & Technology Ceramic College Yixing China

**Keywords:** cobalt‐based catalysts, deep eutectic solvents, ionic liquids, water splitting

## Abstract

The emerging role of ionic liquids (ILs) and deep eutectic solvents (DESs) in the synthesis of cobalt‐based catalysts for water splitting is reviewed. ILs and DESs can serve as solvents and templates due to their unique physicochemical properties. They can efficiently dissolve raw materials and provide a special nucleation and growth environment, obtaining catalysts with novel structures. The designability of ILs and DESs allows for the controlled preparation of catalysts, where they can participate in the reaction as reactants, providing elements such as P, S, N, simplifying the preparation system of cobalt phosphide, sulfide, and nitride. ILs and DESs in catalyst synthesis achieve structural and compositional design, impacting surface adsorption and intermediate stability, allowing precise control over reaction paths and product selectivity. This leads to improved catalytic performance and stability. The review aims to succinctly summarize recent progress and guide researchers in selecting superior solvents for catalyst preparation.

## Graphical abstract

Recent research has focused on using ionic liquids (ILs) and deep eutectic solvents (DESs) to prepare electrocatalysts. These solvents have unique physicochemical properties and are considered green media and functional materials. Cobalt‐based catalysts are commonly used for water splitting. ILs and DESs can improve their adsorption properties and reaction intermediate stability. This review summarizes recent advancements in using ILs and DESs for cobalt‐based catalyst preparation in water splitting, aiming to aid in the design and synthesis of novel and high‐performance electrochemical catalysts.

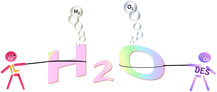

## 1 Introduction

With the ever‐increasing demand for new energy, hydrogen has garnered significant attention as a clean, efficient, and renewable energy source [1, 2]. Electrochemical water splitting has emerged as a viable and efficient approach for producing clean hydrogen and oxygen without any pollution. However, both the hydrogen evolution reaction (HER) and the oxygen evolution reaction (OER) suffer from sluggish kinetics and require high overpotentials [3, 4]. Consequently, the exploration of an efficient electrocatalyst with high electrocatalytic activity has become imperative [5]. In recent years, the study on transition metal‐based catalysts for water splitting has attracted considerable attention.

Cobalt, being widely available in the Earth's crust, ensures a reliable and sustainable supply for the development of catalysts. The electronic structure of cobalt is similar to that of platinum, which suggests that cobalt‐based compounds have the potential to exhibit similar catalytic performance. In addition, they have good chemical stability and can usually resist the erosion of acids, alkalis, and other corrosive media [6, 7, 8]. A large number of cobalt‐based sulfides, phosphides, and nitrides, and so on. have been synthesized by various strategies.

Preparation of cobalt‐based catalysts commonly employs traditional solvents such as water, DMF, and so on. Although the synthesis techniques are relatively mature, the distribution of active sites in the resultant catalysts can be influenced by various factors, such as solvent effects and ionic strength which may lower catalytic performance. Meanwhile, controlling crystal growth using solvents like water is also challenging, making it difficult to manufacture nanocatalysts with specific morphologies and sizes. Additionally, these conventional solvents may lead to evaporation issues, the generation of harmful waste, and pollution risks. Therefore, researchers have been striving to modify the morphology and electronic structure of cobalt‐based catalysts in order to obtain efficient and stable new cobalt‐based electrocatalysts [9]. Employing novel media or reactants is one of effective strategies in this pursuit. Among them, ionic liquids (ILs) and deep eutectic solvents (DESs) have received widespread attention.

ILs are a special type of liquid composed of organic cations and organic or inorganic anions [10, 11]. Unlike traditional solvents such as water and organic solvents, ILs are in a liquid state at room temperature and exhibit unique properties such as low vapor pressure and high thermal stability [12, 13, 14, 15, 16, 17]. As ‘designer liquids’, ILs’ physical and chemical properties can be adjusted by changing the structure and composition of the cations and anions. However, the development of traditional ILs is limited by the complexity and cost of their preparation and purification processes, as well as their potential for environmental pollution [18, 19, 20]. In this context, DESs have attracted attention as an alternative.

In 2003, Abbott et al. discovered that urea and choline chloride (ChCl) could form a liquid at room temperature via simple stirring. They named this new class of solvents as ‘DESs’ [21]. DESs are composed of hydrogen bond acceptors (HBAs) and hydrogen bond donors (HBDs) in specific stoichiometric ratios. The HBAs commonly are quaternary ammonium salts (such as ChCl) and zwitterions, while the HBDs typically include urea, amides, thiourea, and so on*.*. The freezing point of a DES is usually much lower than the freezing points of its individual components, allowing it to exhibit liquid‐like properties at lower temperatures [22, 23, 24]. Owing to their similar properties to ILs, DESs are also referred to as pseudo‐ILs or novel ILs.

ILs and DESs are widely recognized as nontoxic, environmentally friendly, and biodegradable solvents*.* They have been applied in various fields, such as metal extraction, organic compound separation and purification, biocatalytic conversions, and nanoparticle synthesis [25, 26, 27]. IL/DES‐mediated transition metal catalysts have shown excellent performance in the electrochemical water splitting and nitrogen reduction reactions. Compared to water or traditional organic solvents, ILs and DESs have the following advantages in the preparation of inorganic nanomaterials [28, 29, 30, 31, 32]

Solubility: ILs and DESs possess exceptional solubility, rendering them highly proficient in dissolving a multitude of inorganic salts. This remarkable property positions them as unparalleled solvents for catalyst synthesis, as they can effortlessly dissolve metal precursors and nanoparticles.

Size and morphology control: The solubility characteristics of ILs and DESs can be modified to regulate the structure and composition of catalysts. This enables precise control over the size, morphology, and crystal phase of inorganic nanomaterials, facilitating directed synthesis.

Designability: The physical and chemical properties of ILs and DESs can be precisely controlled by adjusting their composition and structure. This allows for the adjustment of parameters such as solvent polarity, acidity/basicity, and ion strength to optimize the synthesis process and properties of nanomaterials. More importantly, it is possible to provide the necessary elements for the desired catalyst to participate in the reaction, reducing reactants and minimizing side reactions.

To compare the differences in the preparation of cobalt‐based electrolysis water catalysts using ILs, DESs, water, and organic solvents as media, including factors such as environmental impact, modulated morphology of catalysts, and so on, a comparative table is outlined (Table 1).

**TABLE 1:** A comparative table outlining the characteristics of ILs, DESs, water, and organic solvents as media.

Comparison items	IL	DES	Water	Organic solvents
Environmental impact	Low	Low to moderate	Very low	Moderateto high
Cost‐effectiveness	High	Moderate	Very low	Low to moderate
Thermal stability	High	Moderate	High (Volatile)	Moderate
Solubility of precursors	High	Moderate	Low	High
Designability	High	High	Non‐adjustable	Non‐adjustable
Safety in operation	High	High	Very high	Low
The ability to modulate morphology of catalysts	High	High	Low	Low
Catalyst performance	High	High	Low	Moderate
Recyclability	High	High	Low	Low

In light of the advancements in this field, we have compiled this mini‐review. It provides a detailed overview of the application of ILs and DESs in the preparation of cobalt‐based catalysts for water splitting. The mini‐review is divided into three parts: the first part provides a popular science introduction to ILs and DESs, highlighting their advantages over traditional aqueous media in the preparation of electrocatalysts; the second part reviews the relevant research on the use of ILs and DESs in the preparation of various types of cobalt‐based compounds in recent years and also summarizes the electrochemical properties of each catalyst (Tables 2, 3, and 4); the third part concludes the review and looks forward to the future development of cobalt‐based electrocatalysts based on ILs and DESs. The aim of this mini‐review is to help researchers understand and keep up with the latest advancements in cobalt‐based catalysts based on ILs and DESs and guide them in selecting superior solvents for catalyst preparation.

**TABLE 2:** HER performance of cobalt‐based catalysts based on IL/DES.

Catalyst	Applied IL/DES	Preparation method	Electrolyte	*η*, mV@Current Density, mA cm^−2^	Tafel slope, mV dec^−1^	Ref.
Co–S/NF	ChCl/EG	Electrodeposition	1 M KOH	124@100	65	[33]
NiCo_*x*_S_*y*_/NF	ChCl/EG	Electrodeposition	1 M KOH	65	62.5	[34]
CoS	ChCl‐EG	Solvent‐thermal method	1 M KOH	105@10	63.5	[35]
N, S,O‐C/Co_9_S_8_	CoCl_2_·6H_2_O/TU	Calcination	1 M KOH	53@10	45.3	[36]
N, S,O‐C/Co_9_S_8_	CoCl_2_ · 6H_2_O/TU	Calcination	1.0 M PBS	103@10	113.7	[36]
N, S,O‐C/Co_9_S_8_	CoCl_2_ · 6H_2_O/TU	Calcination	0.5M H_2_SO_4_	68@10	31	[36]
N, P‐C/NiCoP	[BMIM]PF_6_	Solvent‐thermal method	0.5 M H_2_SO_4_	108@10	68	[37]
N, P‐C/NiCoP	[BMIM]PF_6_	Solvent‐thermal method	1 M KOH	128@10	70	[37]
N, P‐C/NiCoP	[BMIM]PF_6_	Solvent‐thermal method	1.0 M PBS	108@10	82	[37]
CoP/CNT	[MBMG]_2_CoCl_2_Br_2_	Calcination	0.5 M H_2_SO_4_	135@10	58	[38]
CoP/carbon fiber	[BMIM]PF_6_	Inkjet printing	0.5 M H_2_SO_4_	97@10	50.2	[39]
Co_2_P/CNT	[P_66 614_]_2_CoCl_4_	Microwave	0.5 M H_2_SO_4_	135@10	58	[40]
Co_2_P/CNT	[P_66 614_]_2_CoCl_4_	Calcination	0.5 M H_2_SO_4_	150@10	47	[41]
NiCoP	Methyltriphenylphosphonium bromide/EG	Electrodeposition	1 M KOH	93@10	48	[42]
High‐entropy metal phosphide	[P_4444_]Cl/EG/CoCl_2_·6H_2_O/FeCl_3_·6H_2_O/MnCl_2_·4H_2_O/NiCl_2_·6H_2_O/CrCl_3_·6H_2_O	Solvent‐thermal method	1 M KOH	136@10	85.5	[43]
Co_4_N/NC@CC	CoCl_2_⋅·6H_2_O/urea	Calcination	0.5 M H_2_SO_4_	62@10	—	[44]
Co_4_N/NC@CC	CoCl_2_⋅·6H_2_O/urea	Calcination	1 M PBS	98@10	—	[44]
Co_4_N/NC@CC	CoCl_2_⋅·6H_2_O/urea	Calcination	1.0 M KOH	60@10	69.2	[44]

**TABLE 3:** OER performance of cobalt‐based catalysts based on IL/DES.

Catalyst	Applied IL/DES	Preparation method	Electrolyte	*η*, mV@ Current density, mA cm^−2^	Tafel slope, mV dec^−1^	Ref.
Co–S/NF	ChCl/EG	Electrodeposition	1 M KOH	322@100	66.4	[33]
NiCo_*x*_S_*y*_/NF	ChCl/EG	Electrodeposition	1 M KOH	300@20	109	[34]
NiCo_2_S_4_	PEG 200/TU	Solvent‐thermal method	1 M KOH	337@10	64	[45]
CoS	ChCl‐EG	Solvent‐thermal method	1 M KOH	277@10	73.2	[35]
CuCo_2_S_4_	Cobalt(II) bis(n‐butylpyridine) tetrachlorocuprate(II)	Hot injection	1 M KOH	230@10	211	[46]
Fe‐CoNiP	[P(C_6_H_12_)_3_C_14_H_29_][Cl]	Calcination	1 M KOH	355@50	45.0	[47]
High‐entropy metal phosphide	[P_4444_]Cl/EG/CoCl_2_·6H_2_O/FeCl_3_·6H_2_O/MnCl_2_·4H_2_O/NiCl_2_·6H_2_O/CrCl_3_·6H_2_O	Solvent‐thermal method	1 M KOH	320@10	60.8	[43]
P, F‐Ni_1.5_Co_1.5_N	[BMIM]PF_6_	1. Hydrothermal 2.Calcination	1 M KOH	280@10	66.1	[48]

**TABLE 4:** Overall water splitting performance of cobalt‐based catalysts based on IL/DES.

Catalyst	Applied IL/DES	Preparation method	Electrolyte	Potential, V@ Current density, mA cm^−2^	Ref.
Co–S/NF	ChCl/EG	Electrodeposition	30 wt% KOH	1.72 V	[33]
NiCo_*x*_S_*y*_/NF	ChCl/EG	Electrodeposition	1 M KOH	1.57@10	[34]
CoS	ChCl‐EG	Solvent‐thermal method	1 M KOH	1.62@10	[35]
High‐entropy metal phosphide	[P_4444_]Cl/EG/CoCl_2_ · 6H_2_O/FeC_l3_·6H_2_O/MnCl_2_ · 4H_2_O/NiCl_2_ · 6H_2_O/CrCl_3_·6H_2_O	Solvent‐thermal method	1 M KOH	1.78@100	[43]

## 2 The Preparation of Cobalt‐Based Catalysts for Water Splitting Involving ILs and DESs

### 2.1 Cobalt‐Based Sulfide Catalysts

Cobalt‐based sulfides possess excellent conductivity, which can facilitate water splitting at relatively low potentials. This is attributed to the narrow band gap between the valence and conduction bands, aiding electron transitions and the movement of carriers within the material, thereby enhancing the conductivity of cobalt‐based sulfides. Density functional theory studies have indicated that the synergistic effect caused by the bonding between metals and sulfur can optimize the energies associated with water dissociation, hydrogen adsorption/desorption, and the binding of generated oxy‐intermediates [49]. Moreover, cobalt‐based sulfides exhibit good durability and stability, maintaining catalytic activity over extended periods. Sulfides also have the advantages of being low in cost and environmentally friendly. Based on these properties, cobalt‐based sulfides have become materials of great interest in the field of sustainable energy [49]. Common solvents used in the preparation of cobalt‐based sulfides include organic solvents such as DMF, NMP, THF, and ethanol, as well as inorganic solvents like water, hydrochloric acid, and nitric acid. However, the catalytic performance is still not quite ideal. The application of ILs and DESs has brought significant breakthroughs for cobalt‐based catalysts [50].

In the synthesis of cobalt sulfide, thiourea (TU) or S is commonly used as the sulfur source. In ChCl/EG DES system, added TU, a porous Co–S film doped with sulfur (Co–S/NF) was prepared on a nickel foam (NF) electrode using a one‐step electrodeposition method [33]. Unlike traditional media, the unique physicochemical properties of DESs, such as low viscosity and high ionic conductivity, facilitate the diffusion and mass transfer of reactants on the electrode surface, thereby accelerating the electrodeposition process [51]. This DES could form stable Co(II)‐Cl complexes with cobalt ions, which were thermodynamically favored species compared to Co(II)‐S complexes. This characteristic allowed for the control of Co(II)‐S complex formation, directing the growth direction and morphology of Co(II)‐S crystals, which in turn facilitated the regulation of Co–S compound synthesis. Additionally, the special composition of DES confers upon them the ability for self‐assembly, enabling the formation of ordered structures under certain conditions. By precisely regulating these interactions, precise control over the morphology of the products can be achieved. The obtained Co–S/NF fully exposed active sites, demonstrating superior catalytic performance for water splitting. Sulfur doping not only improved charge transfer efficiency but also promoted catalytic reaction kinetics by increasing the electrochemically active surface area and introducing structural defects. Furthermore, during the OER process, sulfur doping also contributed to the formation of oxygen‐deficient Co‐O/OH species, further enhancing its excellent OER performance. In 1 M KOH, the Co–S/NF required overpotentials of 124 and 155 mV to reach current densities of 100 and 500 mA cm^−2^ for HER and overpotentials of 322 and 368 mV for OER. The full water splitting potentials were 1.79 and 2.08 V to reach the same current. In a 30 wt% KOH solution, the Co–S/NF only needed 1.72 V to reach 500 mA cm^−2^ and was able to operate stably for 150 h.

Still using ChCl/EG DES, a mixture of NiCl_2_, CoCl_2_, and thiourea was subjected to electrodeposition, forming a NiCo_*x*_S_*y*_/NF array with S and Co modifications on the surface [34]. The components of DES may interact with the surface of catalyst to form stable surface structures, thereby enhancing the stability of the catalyst during the reaction process. By utilizing the morphological regulation effect of DES, the preparation of superior‐performing bimetallic sulfides with excellent catalytic performance has been realized. NiCo_*x*_S_*y*_/NF was capable of catalyze HER (*η*_10_ = 65 mV, Tafel slope = 62.5 mV dec^−1^), OER (*η*_20_ = 300 mV, Tafel slope = 109 mV dec^−1^), and overall water splitting (achieving a current density of 10 mA cm^−2^ at 1.57 V) in alkaline environments. Compared to monometallic sulfides, bimetallic sulfides excel in water splitting due to their synergistic effect. This effect allows one metal to offer active sites, while the other modulates the electronic structure. This modulation adjusts energy levels, enhancing electron transfer and catalytic activity. Metal interaction provides more active sites, improving reaction rates and catalytic activity. This reduces the energy barrier for water electrolysis. Employing a DES to prepare bimetallic sulfides can provide a conducive environment, which facilitates easier interaction and exchange between the two metal elements in solution.

Thiourea, as a typical HBD, can form DESs with HBAs, creating complexes with unique properties. In the process of preparing cobalt‐based catalysts, there are some differences between using TU‐based DES and TU alone as a sulfur source [52]. Mu et al. [45] successfully prepared a series of sulfides, such as CoS_2_, NiS_2_, and NiCo_2_S_4_, using a simple solvent‐thermal method with polyethylene glycol (PEG) 200/TU DES. This preparation method had two distinct advantages. Firstly, the DES acted as a template and could regulate the morphology of the products. TU‐based DESs provides a unique reaction media that helps regulate reaction conditions and may promote a more effective sulfidation process. DES may offer better control over sulfidation because its composition can be adjusted according to needs, thus optimizing sulfur supply and distribution, which is crucial for the final performance of the catalyst. Moreover, DESs may influence the morphology and crystal structure of the product, which could enhance the activity and stability of the catalyst. For example, in the case of NiCo_2_S_4_, a sea urchin‐like micro/nanostructure with uniform interconnections between nanorods could be formed under the set reaction conditions. The advantages of this strategy include diversity, high solubility, mild conditions, controllability, and interface regulation. Moreover, this DES served as a sulfur source in the reaction, making the reaction system simple, reducing side reactions, and lowering the preparation cost. Additionally, DES has environmental friendliness, as they are generally considered more eco‐friendly alternatives. These characteristics provide us with flexible and designable sulfur sources, offering more possibilities for the preparation of metal. sulfur catalysts. The prepared NiCo_2_S_4_ exhibited OER activity (η_10_  = 337 mV, Tafel slope = 64 mV dec^−1^).

Besides thiourea, Na_2_S_2_O_3_ is also an excellent sulfur source. Mise et al. successfully synthesized monodisperse CoS nanoparticles (≈78 nm) by regulating the dynamic coordination equilibrium between Co^2+^ and S_2_O_3_^2‐^ in the ChCl‐EG DES [35]. This material had a high sulfur content (S/Co = 1.9) and abundant oxygen vacancies, which significantly enhanced its electrochemical activity. In alkaline electrolyte, the CoS/NF electrode exhibited excellent HER and OER performance, with overpotentials as low as 105 and 277 mV respectively, at a current density of 10 mA cm^−2^. In addition, this catalyst only required 1.62 V to drive a current density of 10 mA cm^−2^ in overall water splitting and shows excellent stability.

ILs and DESs can also provide metal sources for the reaction system. Using ILs and DESs as metal sources not only simplifies reaction steps and enhances reaction efficiency and product purity but also allows precise control over the morphology and performance of the products by adjusting concentration of metal ions. Taubert et al. [46] designed a novel IL containing metal ions, with cobalt and copper ions in the anion and N‐butylpyridinium cation. This IL reacted with hexamethyldisilathiane (TMS)_2_S to obtain nanoflower‐shaped CuCo_2_S_4_. Compared to commercially available Pt/C and IrO_2_, nanoflower‐shaped CuCo_2_S_4_ exhibited lower onset potential and better durability. The presence of Co^3+^ sites in CuCo_2_S_4_ was crucial for the OER process, as it can enhance the adsorption of reactive intermediates (such as OH*, O*, OOH*, etc*.*.) generated during the OER process, thereby effectively reducing the difficulty of OER charge transfer.

**FIGURE 1:**
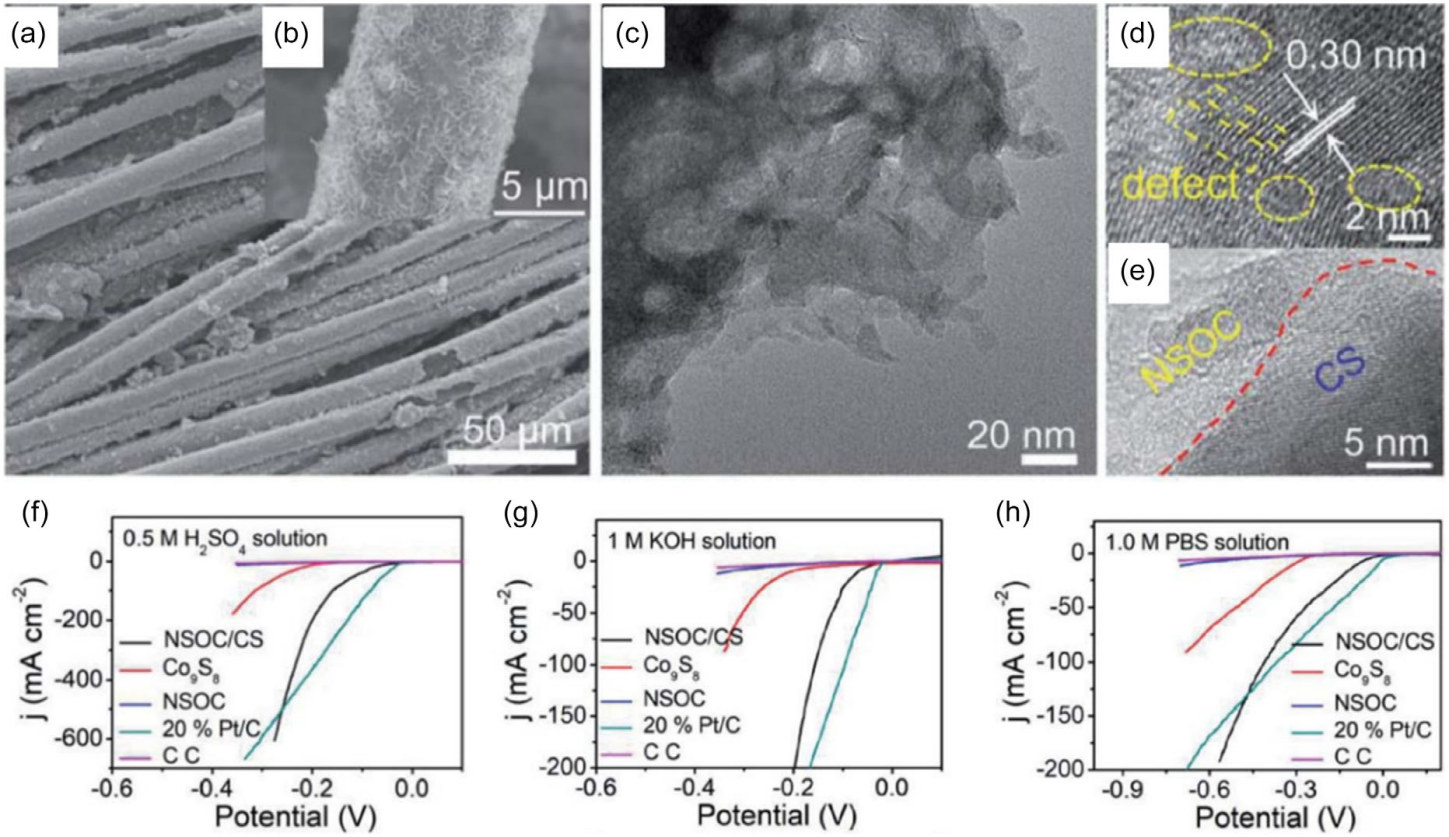
FESEM images of N, S,O‐C/Co_9_S_8_ (a and b), TEM image (c) and HRTEM images (d and e), hydrogen evolution polarization curves of N, S,O‐C/Co_9_S_8_ in 0.5 M H_2_SO_4_ (f), 1 M KOH (g), and 1 M PBS (h)*.* Reproduced with permission from Ref. [36] 2021, Royal Society of Chemistry.

An innovative design is that ILs and DESs can also act as reagents to provide metal and sulfur elements simultaneously through novel design. By coating CoCl_2_·6H_2_O/TU DES on the surface of carbon cloth and using calcination technology, a highly electrocatalytically active defect‐rich N, S, O tri‐doped carbon/Co_9_S_8_ composite (N, S,O‐C/Co_9_S_8_) was prepared (Figures 1a–e) [42]. In this reaction system, both HBD and HBA of DES participated in the reaction. The former acted as the Co source, while the latter acted as the S source. No solvent needed to be discharged, and no other post‐treatment operations were required, which was consistent with the concept of green chemistry and was more conducive to the realization of industrial production. Based on the designability of DES, the composition structure and electrochemical performance of N, S,O‐C/*Co*_9_ *S*_8_ could be reasonably controlled by adjusting the ratio of HBAs and HBDs. In 1.0 M KOH, 1.0 M phosphate buffered solution (PBS) and 0.5 M H_2_SO_4_, the hydrogen evolution overpotential of the composite material reaches 10 mA cm^−2^ at current density when it is 53, 103 and 68 mV, respectively, and the composite material has good stability under all pH values (Figure 1f–h).

Designing ILs and DESs as reactive agents represents a significant advantage over water or conventional organic solvents. This strategy exhibits various benefits in the preparation of catalysts.

1. Precise regulation of the structure and performance of catalysts: The unique molecular structure and solvation effects of ILs and DESs ensure uniform distribution of heteroatoms or metal ions within the materials, which contributes to enhance catalytic activity and electrochemical properties.
2. Simplification and efficiency of the synthesis process: This synthetic strategy eliminates the need for addition of heteroatom sources, not only helping to reduce overall synthesis costs but also minimizing the steps involved in adding these sources to the reaction system. Therefore, this synthetic strategy is economically viable.
3. Environmental friendliness and sustainability: Compared to traditional solvents and templates, ILs and DESs generally have lower toxicity and good biodegradability. They can also be recovered and reused through simple treatments, lowering production costs and waste disposal expenses, aligning with the concept of sustainability.

### 2.2 Cobalt‐Based Phosphide Catalysts

Transition metal phosphides exhibit covalent bonds, delocalized d orbitals, and localized f orbitals. These properties enhance their suitability for the redox reactions in water splitting, boosting the rate and efficiency of water splitting, and facilitating greater production of hydrogen and oxygen. Additionally, transition metal phosphides also demonstrate good durability and stability, maintaining high catalytic activity during long‐term usage [53].

**FIGURE 2:**
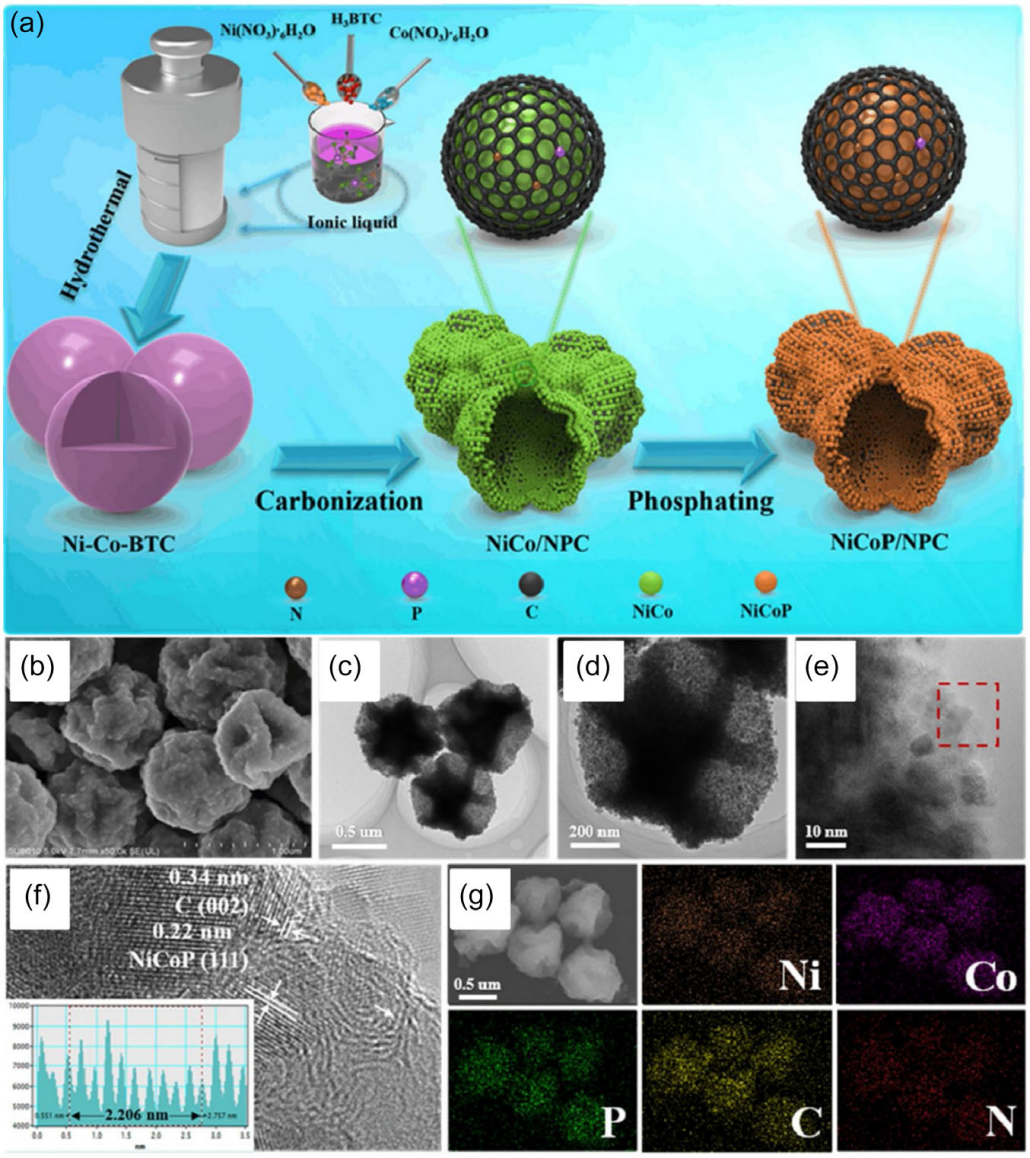
Schematic illustration of the synthesis of N, P‐C/NiCoP (a), SEM images of N, P‐C/NiCoP precursor (b) and N, P‐C/NiCoP (c–e), HRTEM image (f) and elemental distribution map (g) of N, P‐C/NiCoP*.* Reproduced with permission from Ref. [37] 2021, Elsevier.

The performance of phosphide cobalt can be significantly improved through structural modulation. The N, P‐doped carbon/NiCoP (N, P‐C/NiCoP) composite material was prepared using the IL‐assisted method with 1‐butyl‐3‐methylimidazolium hexafluorophosphate ([BMIM]PF_6_) as shown in Figure 2a [37]. ILs can provide stable reaction environments and guide the transformation of reactants in specific directions through specific interactions, thus controlling the morphology and structure of the products. The surface of the prepared material has abundant wrinkles due to the templating effect of IL, and compared to the samples without IL, its surface area increased by 5 times (Figure 2b–g). A larger surface area contributes to improving the electrocatalytic performance of catalysts. ILs were able to enhance the stability of metal complexes, preventing their self‐aggregation during the preparation process. The N, P‐C/NiCoP could undergo HER in acidic, alkaline, and neutral electrolytes. The *η*_10_ values of N, P‐C/NiCoP were 108 mV (under acidic conditions), 128 mV (under alkaline conditions), and 108 mV (under neutral conditions), respectively. Wang et al. reported a method for the in situ synthesis of iron‐doped transition metal phosphides (Fe‐CoNiP‐C/NF) on NF via a direct phosphidation strategy using ILs as precursors [47]. The catalyst was prepared by pyrolysis at 450°C with trihexyltetradecylphosphonium chloride ([P(C_6_H_12_)_3_C_14_H_29_][Cl]) as the phosphorus source, combined with FeCl_3_ and CoCl_2_, forming Fe‐CoNiP nanoparticles embedded in carbon layers. This Fe‐CoNiP‐C exhibited excellent oxygen OER performance in 1 M KOH, requiring only overpotentials of 355 mV and 424 mV to achieve current densities of 50 and 100 mA cm^−2^, respectively, and could stably operate at high current densities for more than 30 h.

It can be anticipated that ILs and DESs can be designed as precursors for cobalt phosphide. This allows for modulation of the microenvironment of the metal or heteroatom sources, thereby controlling the structure and uniformity of active sites of catalyst, which in turn affects the performance. Meanwhile, The environmental compatibility of ILs and DESs can minimize the impact of the reaction system on the environment. Typically, metal elements are designed as anions and incorporated as components of ILs. For example, N, N‐Bis(4‐(methoxycarbonyl)benzyl)‐N‐methyl‐D‐glucamine dibromodichlorocobaltate(II) IL ([MBMG]_2_CoCl_2_Br_2_) can be an excellent source of metal. This design allows for precise manipulation of the chemical environment surrounding cobalt, which is crucial for obtaining desired properties in the final product. This precision can lead to the formation of specific phases or structures of cobalt phosphate that are not achievable through traditional methods. It was mixed with carbon nanotubes (CNTs) and subjected to a low‐temperature phosphidation process at 300°C, successfully yielding CoP/CNT [38]. The phosphorization process was characterized by FTIR, Raman spectroscopy, XPS, and XRD, etc*.*. and found that CoCl_2_Br_2_^2‐^ reacted with NaH_2_PO_2_ to form CoP, while [MBMG]^2+^ transformed into amorphous carbon. The in situ introduction of amorphous carbon improved the conductivity of CoP and enhanced electron transfer ability. Experimental results confirmed that CoP/CNT is a highly active HER electrocatalyst, with a low onset potential of only 55 mV, *η*_10_ of 135 mV, and a Tafel slope of 58 mV dec^−1^. IL‐derived prepared strategy would make it more environmentally friendly compared to other methods that might involve harsh chemicals or generate hazardous waste. While the initial cost might be high due to the complexity of synthesizing [MBMG]_2_CoCl_2_Br_2_, its ability to provide high yields and purity under milder conditions might offset these costs in large‐scale production, making it a cost‐effective option in the long run.

Traditional phosphorus sources commonly include trioctylphosphine oxide, NaH_2_PO_4_, P, and so on. However, ILs containing P element can also serve as phosphorus sources under appropriate conditions, allowing phosphorus to be released from IL and react with cobalt. A prominent advantage of this synthetic strategy is enhanced control over the morphology of catalyst [54]. Using a simple inkjet printing technique, the combination of [BMIM]PF_6_ IL with metal salt solution was utilized to prepare metal phosphides (CoP, MoP, FeP, NiP)/carbon fiber composites with excellent HER performance across the entire pH range [39]. The IL served as both a phosphorus source and a carbon source. As a result, an in situ carbon nanolayer was obtained, which exposed abundant catalytic active sites during the HER process and improved the electron transfer rate. The obtained in situ carbon nanolayer also prevented the corrosion of transition metal. phosphide nanoparticles, enhancing the stability of the materials. Taking the example of CoP/carbon fiber composite, in 0.5 M H_2_SO_4_, this catalyst achieved an overpotential of 97 mV to reach a current density of 10 mA cm^−2^. The Tafel slope was measured to be 50.2 mV dec^−1^. Integrating the phosphorus source into an IL offers greater flexibility in the design and functionalization of catalysts. By choosing different ion combinations to change their polarity, viscosity, density, and other physical and chemical properties, it is possible to achieve a controlled synthesis of the catalyst. Meanwhile, ILs have unique solvent capabilities that can dissolve substances that are difficult to handle with traditional solvents, expanding their range of applications. Moreover, compared to many organophosphorus compounds, P‐ILs have lower vapor pressures.

Metal phosphides can also be obtained by using IL cation as a P source in the reaction. In this study, our research group utilized tetrabutylphosphonium chloride ([P_4444_]Cl) IL as the medium and template to prepare highly catalytic nickel‐based catalysts. The work demonstrated that quaternary ammonium salts can serve as excellent phosphorus sources [55]. Based on this work, Li research group [40, 41]. used [P_66 614_]_2_CoCl_4_ as a source of phosphorus and cobalt and prepared Co_2_P/CNT through microwave or calcination methods through mixing with CNTs. The addition of CNTs can enhance conductivity and facilitate the formation of cobalt phosphide.In acidic solutions, Co_2_P/CNT exhibited excellent HER activity. Taking the example of Co_2_P/CNT synthesized by the microwave method, its onset overpotential was as low as 80 mV, and the Tafel slope was measured to be 58 mV dec^−1^ [40]. Certainly, using ILs as a phosphorus source also has some limitations. The synthesis of P‐ILs may be more complex and costly than traditional phosphorus sources. P‐ILs may be less readily available and have handling difficulties compared to traditional phosphorus sources, limiting their widespread application. Additionally, while P‐ILs are generally considered environmentally friendly, certain P‐ILs may have unknown toxicity issues that require further study and evaluation.

DESs have also attracted attention in the preparation of metal. phosphides. Wang et al.*.* [42] used methyltriphenylphosphonium bromide/EG DES as the electrolyte and employed a constant potential method to electrodeposit nickel cobalt phosphide (NiCoP) thin films onto carbon cloth surfaces. The formation of this film followed a diffusion‐controlled three‐dimensional instantaneous nucleation and growth mechanism, where the film formation was mainly controlled by the diffusion process. Firstly, molecules diffused in the solution or gas phase, and then under appropriate conditions, three‐dimensional instantaneous nucleation occurred, where molecules aggregated to form primary nuclei. Subsequently, these primary nuclei grew through processes such as surface diffusion and material adsorption, ultimately forming a complete film. The NiCoP film exhibited extremely high HER activity, with an overpotential of 93 mV (*η*_10_) and a Tafel slope of 48 mV dec^−1^ in 1 M KOH.

High‐entropy metal phosphides refer to mixtures that contain five or more different metal elements. These multicomponent compositions can be tailored based on the Sabatier principle, which states that the chemical activity of a catalyst is highest when the interaction between reactant molecules and the catalyst surface reaches an ideal equilibrium. By adjusting the composition, the optimal adsorption state of reaction intermediates can be achieved, thereby further enhancing the electrocatalytic activity for water splitting [56]. Mu et al. [43] designed and synthesized a quaternary ammonium‐based DES consisting of [P_4444_]Cl/EG/five equimolar hydrated metal chlorides (CoCl_2_ *·*·6H_2_O, FeCl_3_ *·*·6H_2_O, MnCl_2_ *·*·4H_2_O, NiCl_2_ *·*·6H_2_O, CrCl_3_ *·*·6H_2_O). In this system, [P_4444_]Cl and the hydrated metal chlorides served as HBA, while EG acted as HBD. The DES, which acted as both the metal source and phosphorus source, enabled in situ phosphidation under inert atmosphere to obtain high‐entropy metal phosphides. The template effect of DES help to precisely control the morphology and structure of the catalyst, which is crucial for its performance. The selection and proportion of different metal chlorides in DES directly affect the multicomponent composition of the final catalyst, thereby influencing its electronic structure and active sites for catalysis. This high‐entropy catalyst exhibited dual catalytic functions, efficiently catalyzing the HER (*η*_10_ of 136 mV, Tafel slope of 85.5 mV dec^−1^) and OER (*η*_10_ of 320 mV, Tafel slope of 60.8 mV dec^−1^). The experimental results suggest that the tunability of DES offers a promising strategy for designing high‐entropy metal phosphide catalysts. However, the feasibility and sustainability of this approach in practical applications still require further validation through more research. Firstly, this strategy involves a certain degree of synthetic complexity. The synthesis of DES may involve complex steps requiring precise control over the proportions of various components and reaction conditions, and the solubility of the components in DES determines the distribution of metals and phosphorus sources in the reaction system, which can affect the uniformity and purity of the catalyst. This might increase production costs and process complexity. Additionally, the recovery and reuse of DES after the reaction may be challenging, which could affect their practicality.

### 2.3 Cobalt‐Based Nitride Catalysts

Transition metal nitrides are formed by filling the voids in the metal lattice with nitrogen atoms. N can alter the *d*‐band of metal, giving the nitrides catalytic properties similar to noble metals and thus enhancing catalytic activity. The other characteristic of metal nitrides is that they typically have abundant active sites on their surfaces, such as bonding sites between nitrogen and metal atoms. These sites can provide active centers required for catalytic reactions. These active sites offer high electron transfer rates and great catalytic activity, promoting the generation of oxygen and hydrogen during the process of water electrolysis [57].

However, the synthesis of metal nitrides is relatively difficult and complex. The commonly used method involves the nitridation reaction using NH_3_. This preparation typically requires high temperature and high‐pressure conditions, as well as specific reaction systems and atmospheres. Zhang and Wang synthesized P‐ and F‐doped cobalt‐based nitride (N, P‐Ni_1.5_Co_1.5_N) using a simple strategy [48]. They injected an appropriate amount of [BMIM]PF_6_ into a Co^2+^:Ni^2+^ solution with a mass ratio of 1:1 to obtain the precursor. The phosphorus and nitrogen atoms played a role in promoting the primary nitridation reaction and introducing phosphorus into the precursor in [BMIM]PF_6_. The introduction of IL as a template agent may simplify the synthesis process and reduce the required temperature and pressure conditions, making the synthesis milder. Moreover, IL serves as both a solvent and a nitrogen source, simultaneously promoting the doping of phosphorus into the precursor structure. Subsequently, the precursor was subjected to a secondary nitridation process under an NH_3_ atmosphere to obtain N, P‐Ni_1.5_Co_1.5_N. The mixing of IL with Co^2+^ and Ni^2+^ solutions may facilitate the formation of ordered precursor structures, which is beneficial for subsequent nitridation reactions. By adjusting the amount of IL and the ratio of metal ion, the composition of the precursor can be precisely controlled, thereby optimizing the performance of the final material. Owing to the dual nitridation and doping of heteroatoms, P, F‐Ni_1.5_Co_1.5_N exhibited high OER performance, with an overpotential as low as 280 mV to achieve a current density of 10 mA cm^−2^ and a Tafel slope of 66.1 mV dec^−1^. It should be noted that the selection and amount control of [BMIM]PF_6_ need to be cautious in this work. Additionally, the universality of this strategy deserves further exploration.

**FIGURE 3:**
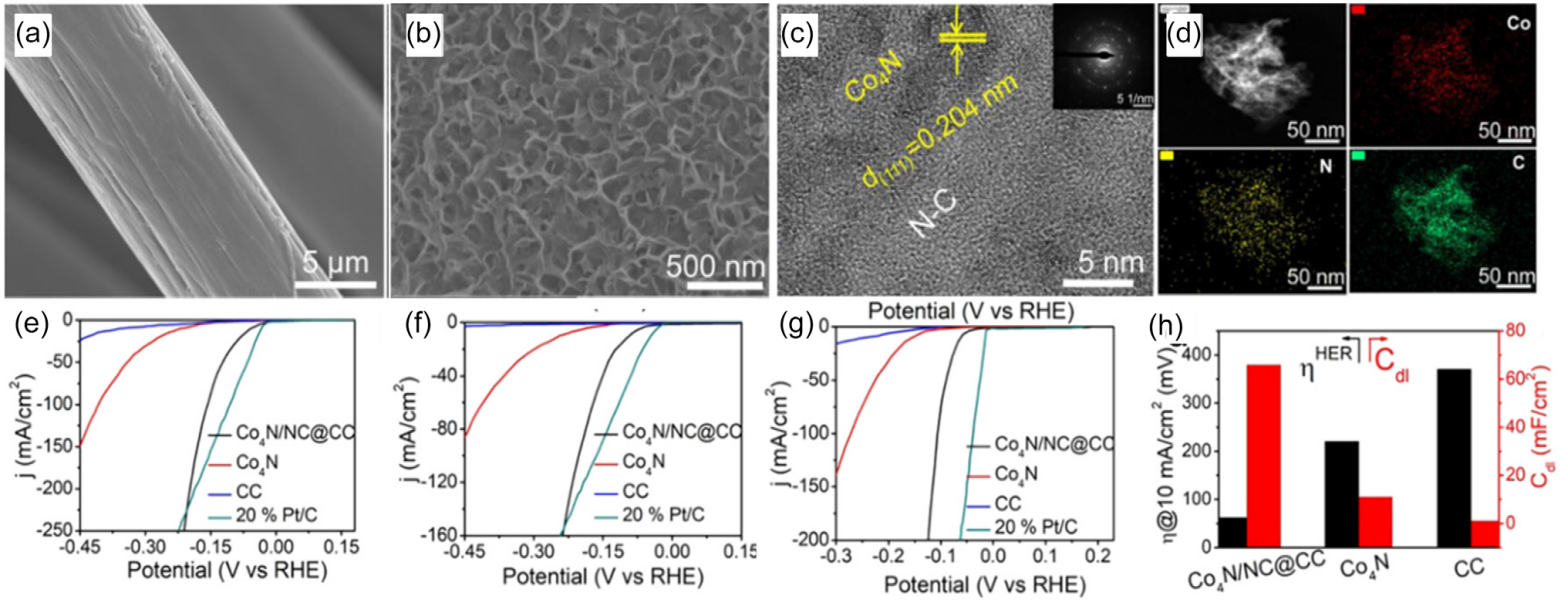
(a) SEM image of pristine carbon fiber; Morphology characterization of Co_4_N/NC@CC; (b) SEM image grown on carbon fiber; (c) HRTEM image of Co_4_N/NC; The inset in (c) shows SAED pattern; and (d) STEM image of Co_4_N/NC and the corresponding elemental mapping images of Co, N, and C; (e) LSV curves of different catalysts (Co_4_N/NC@CC, Co_4_N, CC, 20% Pt/C) for HER in 1.0 M KOH; (f) LSV curves in 1.0 M PBS; (g) LSV curves in 0.5 M H_2_SO_4_; and (h) the comparison of HER overpotential (at 10 mA/cm^2^) and C_dl_ of Co_4_N/NC@CC, Co_4_N, CC. Reproduced with permission from Ref. [44] 2023, Elsevier.

Recent studies have shown that it is possible to use DES as a nitrogen source to prepare nitrides instead of NH_3_. Wang et al. [44] directly coated CoCl_2_ · 6H_2_O/urea DES on the surface of carbon cloth (Figure 3). Through high‐temperature calcination, N‐doped carbon (NC) composite Co_4_N ultrathin nanosheets (Co_4_N/NC) were generated on the surface of carbon cloth. The carbon cloth as a carrier provided stable support and good electronic conduction pathways. The urea in DES acted as a nitrogen source. During the high‐temperature calcination process, urea decomposed, releasing nitrogen atoms that could react with Co_4_N to form N‐doped carbon layers. This strategy simplified the synthesis process of composite materials and improved their uniformity and adhesion. The strong interaction between Co_4_N and NC could significantly accelerate electron transfer at the interface, and the ultrathin nanostructure of Co_4_N/NC could increase the catalytic active sites, thereby effectively improving the catalytic performance of Co_4_N/NC. In the HER process, Co_4_N/NC achieved a current density of 10 mA cm^−2^ with overpotentials of only 62, 98, and 60 mV in alkaline, neutral, and acidic electrolytes, respectively. Based on urea‐based DES as a nitrogen source, it offers a simple method to control the degree and distribution of nitrogen doping, which is crucial for enhancing the electronic conductivity and catalytic activity of the catalysts.

The key parameters of cobalt‐based catalysts based on IL/DES for HER, OER, and water splitting are summarized in Tables 2‐4, including ILs or DESs involved in catalysts, the preparation methods, current densities, and overpotentials. It is shown that various cobalt‐based catalysts, such as cobalt sulfides, phosphides, and nitrides, can be prepared using IL/DES. These catalysts not only exhibit excellent HER catalytic capabilities but also demonstrate superior activity for OER and overall water splitting. These observations highlight the significance of exploring the applications of IL/DES in the field of electrocatalysis.

## 3 Summary and Outlook

Recent research progress indicates that the utilization of ILs and DESs for the preparation of cobalt‐based catalysts holds great potential in the field of water splitting. ILs and DESs, as green media and functional materials, possess unique physicochemical properties that can influence the adsorption characteristics of catalyst surfaces and the stability of reaction intermediates, thereby enhancing catalytic activity and stability. Additionally, ILs and DESs serve as solvents, templates, enabling better dissolution of catalyst precursors and providing a special nucleation and growth environment to obtain structurally novel catalysts. A highly appealing synthesis strategy is that ILs and DESs serve as the reactive reagents besides as the reaction media. When serving as reactants, they can participate in reactions, introducing elements such as P, S, N, and so on, thus simplifying the preparation processes of cobalt phosphide, sulfide, and other catalysts, reducing emissions, and realizing atom economy [58, 59].

However, there are still some limitations in the utilization of ILs and DESs for the preparation of cobalt‐based catalysts. The selection and design of ILs and DESs require more in‐depth research. While emphasizing the composition of ILs and DESs, it is also necessary to pay more attention to other parameters, including viscosity, electrical conductivity, hygroscopicity, thermal stability, electrochemical stability, recyclability, potential toxicity, and so on. These factors directly influence the performance and safety of catalysts. Viscosity affects the mobility of molecules within media and the diffusion rate of reactants. Viscosity of media should be moderate to ensure it does not impede reaction kinetics while maintaining the stability of the catalyst particles. Additionally, the solvent's electrical conductivity must be considered. Good conductivity is particularly important for electrocatalysts as it affects the efficiency of charge transfer. Choosing a solvent with high electrical conductivity can enhance electron transfer rates, thereby increasing the activity of the catalyst. Furthermore, the thermal stability and electrochemical stability of ILs and DESs are crucial. The solvent should remain stable over a wide potential range to avoid unnecessary side reactions or decomposition in catalytic reactions. In today's advocacy of green chemistry, the recyclability and potential toxicity of media must also be considered. Recyclable solvents help reduce costs and environmental impact. The ease of separation and reuse of solvent is especially important for industrial applications. And it is essential to choose solvents with low toxicity to ensure safety.

ILs are generally more expensive than traditional solvents [16], whereas DESs often exhibit better costeffectiveness due to their low‐cost raw materials and simple preparation [21, 23, 27]. The main reasons for the higher cost of ILs are that their complex synthesis processes and the need for high‐purity raw materials and special reaction conditions, posing challenges for large‐scale applications. The technical difficulties involved in their preparation and purification require specialized knowledge and experimental skills, along with precise control of reaction conditions to prevent side reactions and degradation of catalyst performance. Although some ILs and DESs are recyclable, recovery rates are often low, and the recycling process can be intricate, as they may form difficult‐to‐separate mixtures with catalysts or reaction products, or degrade during recovery, losing their original properties. There is also an insufficient assessment of their environmental impact; while they may have lower toxicity and environmental risks under certain conditions, long‐term use and substantial emissions could potentially affect the environment, necessitating further research and evaluation.

Therefore, future research should prioritize targeted strategies to overcome these limitations. At the molecular level, efforts should focus on designing multifunctional IL/DES systems that integrate the roles of solvent, template, and dopant source, while optimizing physical properties like viscosity and conductivity through structural engineering. To address cost and recyclability challenges, the development of low‐cost feedstocks (e.g., from biomass), streamlined synthesis, and efficient separation protocols are crucial for enhancing solvent recovery and cycling stability. For broader applications, customizing IL/DES formulations for demanding conditions such as seawater electrolysis will be essential to improve corrosion resistance and overall durability. Ultimately, by refining the selection and design of ILs/DESs and their corresponding catalyst synthesis routes, we can accelerate the industrial adoption of high‐performance cobalt‐based catalysts. We hope this review offers valuable insights and aids in the rational design of novel, high‐efficiency electrocatalysts.

## Funding

This research was supported by the Jiangsu Provincial University Philosophy and Social Science Research General Project (No. 2024SJYB0716) and the Jiangsu Provincial Qinglan Project (Talent Project).

## Conflicts of Interest

The authors declare no conflicts of interest.

## Data Availability

All data analysed during this study are included in this published article.
